# A Prospective Observational Study to Evaluate the Factors Affecting the Dermatology Life Quality Index in Patients With Hidradenitis Suppurativa

**DOI:** 10.7759/cureus.35510

**Published:** 2023-02-26

**Authors:** Aastha Menon, Pawan Gupta, Ravleen K Suri, Vaishali Thakare, lily Dubey, Sharmila Patil

**Affiliations:** 1 Dermatology, Dr. D. Y. Patil Hospital, Navi Mumbai, IND; 2 Dermatology, Bundelkhand Medical College, Sagar, IND; 3 Pharmacology, Dr. D. Y. Patil Hospital, Navi Mumbai, IND; 4 Pharmacology, Bundelkhand Medical College, Sagar, IND

**Keywords:** quality of life, disease site, hurley's staging, hidradenitis suppurativa, dermatology life quality index

## Abstract

Background: Hidradenitis suppurativa (HS) is a chronic, relapsing inflammatory skin disease that significantly affects the quality of life of patients. Multiple factors affect the disease's course and severity. HS is a debilitating disease and often recalcitrant to treatment, resulting in a deterioration of quality of life; hence, there is a need to evaluate the factors affecting the quality of life in patients with HS.

Objectives: The objective of the study was to evaluate the various demographic and disease-related factors that affect the quality of life of patients with HS.

Materials and methods: This is a prospectively scored questionnaire-based observational study. Data from 30 patients with HS were analyzed for the association of disease-related factors like Hurley’s staging, site, duration, past history, and comorbidities with the Dermatology Life Quality Index (DLQI).

Results: A statistically significant relationship was found between DLQI and Hurley staging (p=0.000). The most common sites involved were the axilla and inguinal regions. Among the sites involved, the neck (p=0.002), abdomen (p=0.002), back (p=0.002), thighs (p=0.042), and gluteal (p=0.000) regions have a statistically significant association with DLQI. Prior histories of rheumatoid arthritis, scarring, surgery, lymphadenitis, and pilonidal sinus showed a statistically significant association with DLQI.

Conclusion: The disease severity significantly hampers the quality of life of patients with HS. The disease site and presence of other comorbidities also influence the outcome. Our study will help healthcare providers better understand and fulfill the needs of patients suffering from HS.

## Introduction

Hidradenitis suppurativa (HS), also called acne inversa, is a chronic relapsing inflammatory skin disease with different phenotypes and varied severity [1]. The lesions include painful nodules and abscesses, draining tracts, and fibrotic scars. These usually occur in intertriginous areas and areas rich in apocrine glands. As per the previous studies, the prevalence of HS is 1-4%, and the most affected gender is female [2]. The pathogenesis of HS is not certainly understood, but the follicular part of the folliculopilosebaceous unit has been implicated. Among the most common are hair follicles of the axillary, groin, perianal, perineal, and inframammary locations. The clinical picture can range from a few pimples, acne cysts, or boils to malodorous oozing abscesses, sinus tract formation, and scarring [3]. Patients suffering from HS have a negative psychosocial impact because of its sensitive locations, odor, and scarring [4]. Management of this disease depends on its severity and includes antibiotics in the form of topical and systemic administration, hormone therapy, immune modulators, and surgery. Despite the availability of various methods, it has been unsatisfactory and difficult to control this disease [5].

HS involves extensive areas. It manifests as difficulty in everyday activities due to pain, purulent discharge, and malodorous oozing, leading to social embarrassment and a significant impact on quality of life [6].

There is a lacuna in the knowledge of this disease, resulting in delays in the correct diagnosis and management of patients because of a lack of specific diagnostic criteria. It is important to document the quality of life and damage caused by patients with HS, as in India, healthcare policy planning is patient-oriented [7].

This study aims to improve knowledge pertaining to the risk factors for Hidradenitis suppurativa by correlating the demographic features, duration of disease, and comorbidities with the Hurley staging system. We also tried to find a correlation between the Dermatology Life Quality Index (DLQI) score and the Hurley staging system. Hurley's staging is as follows: stage 1 - the presence of abscesses with no sinus tracts and no cicatrisation; stage 2 - recurrent abscesses with tract formation and cicatrisation; and stage 3 - diffuse or near-diffuse involvement, multiple interconnected tracts, and abscesses across the entire area.

## Materials and methods

This was a prospective study conducted in the dermatology outpatient department of Dr. D. Y. Patil Hospital, Navi Mumbai, Maharashtra, India. We initiated the study after receiving approval from the Institutional Ethics Committee for Biomedical and Health Research (Reference no. DYP/IECBH/2022/009).

Thirty patients with HS were included in the study after confirmation of the clinical diagnosis by a dermatologist. The data collected from the individual subject were demographic details like age, gender, height, weight, and body mass index (BMI); disease details like disease duration, site, number, and morphology of the lesions; Hurley's staging system; and the DLQI. DLQI was assessed by subjecting the patients to a questionnaire via a physician-assisted interview after taking their informed consent. DLQI consists of 10 questions. It is a simple and validated questionnaire used to report the impact of the dermatological condition on the quality of life of the patients over the last week, as shown in Table 1. The DLQI score was calculated and noted on the questionnaire sheets of the respective patients. The patient’s disease severity was graded depending on the range of DLQI scores as shown in Table 2.

**Table 1:** Dermatology Life Quality Index (DLQI) questionnaire Key: Very much: 3, A lot: 2, A little: 1, Not at all: 0, Not relevant: 0

S. No.	Questions	Scoring
1.	Did you experience itchiness, pain, soreness, or stinging over the last 7 days?	3/2/1/0
2.	Over the last week, how embarrassed or self-conscious have you been because of your skin?	3/2/1/0
3.	Do you feel your skin has affected your daily activities like shopping, household chores, and looking after your family over the last 7 days?	3/2/1/0
4.	To what extent has this disease affected your choice of clothes over the last 7 days?	3/2/1/0
5.	To what extent has this disease affected any leisure activities you wanted to participate in over the last 7 days?	3/2/1/0
6.	Has the disease caused you to avoid sports activities over the last 7 days?	3/2/1/0
7.	To what extent has your skin prevented you from being able to study or work over the last 7 days ?	3/2/1/0
8.	Has your skin created problems with your relatives or your partner in the last 7 days?	3/2/1/0
9.	Has your skin caused you sexual difficulties in the past 7 days?	3/2/1/0
10.	Has the treatment for the disease taken up too much of your time or caused a mess in the last 7 days?	3/2/1/0

**Table 2:** Dermatology Life Quality Index (DLQI) score with grading

DLQI score	Severity grading
0–1	No effect on patient's life
2–5	Minimal effect on patient's life
6–10	Moderate effect on patient's life
11–20	Very large effect
21–30	Extremely large effect

Statistical analysis

Statistical analysis was done using IBM SPSS Statistics v 26 (IBM Corp., Armonk, NY). The Mann-Whitney u test or student’s t-test were used to evaluate quantitative variables depending on their non-parametric or parametric distribution, respectively. For evaluating qualitative data and the association between the variables, the chi-squared test and Spearman’s test were used, respectively. The difference in total DLQI between patients with different HS severity levels according to Hurley stages was assessed using the Kruskal-Wallis one-way analysis test. A P-value of ≤ 0.05 was considered statistically significant.

## Results

The study included 30 patients with Hidradenitis suppurativa ranging in age from 18 to 63 years old. The demographic details of the patients are presented in Table 3, and the disease characteristics of the patients are in Table 4.

**Table 3:** Demographic details of the study participants N: number of study participants

Demographic parameters	Mean ± SD or N (%)
Age (years)	30 ± 10.79
Weight (kg)	67.73 ± 12.59
Height (m)	158.9 ± 8.47
Body mass index (BMI)	26.54 ± 4.56
BMI category	Category 0 - 0 (0%), category 1 - 10 (33.3%), category 2 - 13 (43.3%), category 3 - 6 (20%), category 4 - (3.3%)
Gender	Males - 17 (56.7%), females - 13 (43.3%)

**Table 4:** Disease characteristics of the study participants

Disease characteristics	Number (percentage)
Hurley’s staging	Category I- 16 (53.3%), Category II- 11 (36.7%), Category III- 3 (10%)
Dermatology Life Quality Index (DLQI)	Category I- 0(0%), Category II- 6 (20%), Category III- 15 (50%), Category IV- 7 (23.3%), Category V- 2 (6.7%)
Location of the lesion	
Right axilla	22 (73.3 %)
Left axilla	22 (73.3%)
Neck	1 (3.3%)
Abdomen	1 (3.3%)
Buttocks	4 (13.3%)
Inguinal	6 (20%)
Back	1 (3.3%)
Thighs	2 (6.7%)
Gluteal	3 (10%)
Inframammary	1 (3.3%)
Associated comorbidities and past history	
Acanthosis nigricans	4 (13.3%)
Acne grade 2	9 (30%)
Folliculitis	1 (3.3%)
Pilonidal sinus	1 (3.3%)
Alcoholism	1 (3.3%)
Smoking	1 (3.3%)
Pityriasis versicolor	1 (3.3%)
Diabetes mellitus	5(16.7%)
Hypothyroidism	2(6.7%)
Tinea cruris	1 (3.3%)
Dyslipidemia	1 (3.3%)
Acrochordon	1 (3.3%)
Hypertension	2(6.7%)
Rheumatoid arthritis	1 (3.3%)
Scar/contracture	2(6.7%)
Dowling Degos disease	1 (3.3%)
Past history	
Surgery	2(6.7%)
TB lymphadenitis	1 (3.3%)
Pilonidal sinus	1 (3.3%)

A statistically significant relationship was found between DLQI and Hurley staging (p = 0.000), indicating decreased quality of life with increasing severity of disease, supporting the finding. Lesions were most commonly located at the axilla and inguinal regions, but no significant correlation was seen with DLQI. Among the sites involved, the neck (p=0.002), abdomen (p=0.002), back (p=0.002), thighs (p=0.042) and gluteal (p=0.000) regions have a statistically significant association with DLQI. Prior history of rheumatoid arthritis (p=0.002), scar (p=0.042), surgery (p=0.042), lymphadenitis (p=0.002), and pilonidal sinus (p=0.002) showed statistically significant association with DLQI.

## Discussion

Out of the total of 30 patients with HS, 17 (56.7%) were males, which confirms the male preponderance of the disease. This finding may be different from those reported by previous studies due to the smaller sample size in our study [8,9]. The most common sites involved were the right and left axilla (73.3%), followed by the inguinal (20%), buttocks (13.3%), and gluteal (10%); these data are comparable to studies conducted in the past [7,8]. The majority of patients in our study, that is, 16 (53.3%), were categorized as Hurley category I. This is different from the findings of studies done by Kamat et al. and Alamri et al., where Hurley's stage 3 disease was found to be most prevalent. The mean DLQI score was 9.47 ± 5.69, which indicates a significant impairment in the quality of life of patients suffering from HS. The mean DLQI of our study population is comparably low to the mean DLQI of studies done in the past by Alamri et al. (15.39 ± 8.37), Matusiak et al. (12.7 ± 7.7), and Schneider-Burrus et al. (13.18 ± 0.37) [8-10]. This can be attributed to the higher number of patients with Hurley stage 3 disease in the above-mentioned studies.

A statistically significant relationship was found between DLQI and Hurley staging (p = 0.000), indicating the decreasing quality of life with increasing severity of the disease, supporting the findings of Afsaneh et al. and Vassiliki et al. [2,11]. Among the sites involved, neck (p=0.002), abdomen (p=0.002), back (p=0.002), thighs (p=0.042) and gluteal (p=0.000) regions have a statistically significant association with DLQI, which is quite similar to the findings of Krajewski et al. [3]. This could be explained by the fact that the above-mentioned sites cause greater hindrances in daily life activities. The most commonly affected sites like the axilla and inguinal area were not significantly correlated to DLQI. The reason may be that these sites are unexposed. Prior history of rheumatoid arthritis (p=0.002), scarring (p=0.042), surgery (p=0.042), lymphadenitis (p=0.002), and pilonidal sinus (p=0.002) showed statistically significant association with the DLQI, suggesting a higher impact on the quality of life of these patients.

Interestingly, no significant correlation was found between DLQI and demographic parameters like age, gender, weight, or body mass index supporting the fact that the intensity of disease plays a major role in the impairment of quality of life, unlike the findings in the study done by Krajewski et al. [3]. No correlation was found between obesity and the smoking status of patients with DLQI, even though both factors are related to the pathogenesis of HS.

When compared to other chronic skin diseases, the DLQI of HS was found to be higher than most skin disorders like psoriasis, lichen planus, bullous pemphigoid, and vitiligo [12,13]. Hidradenitis suppurativa is not a life-threatening disease, but it has a considerable impact on the deteriorating quality of life. Our study will help in planning healthcare policies according to the disease burden.

A major limitation of our study was the small sample size, which can result in varied outcomes of statistical analysis that do not correlate with the results of previous studies. We suggest considering variables like type and duration of treatment to be included in future studies as they can significantly affect the DLQI of HS patients.

## Conclusions

Hidradenitis suppurativa is a chronic, debilitating skin condition. Our study shows a significant impairment in the mental and physical quality of life of patients suffering from the disease. We are in need of novel and improved treatment modalities for the treatment of HS to reduce the burden of the disease. We suggest a holistic approach to the treatment of HS patients, which should include psychological counseling along with exploring all possible treatment options, including biologics, according to individual patient needs.
